# The Expression of Proteases and the Oligopeptide Transporter *PepT1* in the Yolk Sac Membrane, Proventriculus, and Small Intestine During the Development of *Anas platyrhynchos domestica* Embryo

**DOI:** 10.3390/biology13120989

**Published:** 2024-11-29

**Authors:** Seba Jamal Shbailat, Ibtisam Omar Aslan

**Affiliations:** Department of Biology and Biotechnology, Faculty of Science, Hashemite University, P.O. Box 330127, Zarqa 13133, Jordan

**Keywords:** *Anas platyrhynchos domestica*, yolk sac membrane, proventriculus, duodenum, jejunum, ileum, cathepsin B, cathepsin D, aminopeptidase N, the oligopeptide transporter PepT1

## Abstract

The role of the yolk sac membrane (YSM) and digestive tract in the processing of egg yolk proteins during embryogenesis is unexplored in the duck *Anas platyrhynchos domestica*. Here, we investigated in the duck embryo the function of the YSM, proventriculus, and small intestine in protein digestion and uptake. We tested the expression of aminopeptidase N (*APN*) and the oligopeptide transporter *PepT1* as well as the expression of cathepsin B (*CTSB*) and cathepsin D (*CTSD*) lysosomal genes in the YSM during incubation days 12, 14, 16–18, 20, 22, 24, 26, and 28 (the day of hatch). Also, we examined embryonic duck pepsinogen (*EDPg*) expression in the proventriculus and *APN* and *PepT1* expression in the small intestine. In the YSM, *CTSD* expression was weak compared to that of *CTSB*, and the expression of *CTSB*, *APN*, and *PepT1* reached its maximum on day 24 and decreased afterwards. In the proventriculus, *EDPg* expression peaked on days 17 to 20 and decreased thereafter. The *APN* and *PepT1* expression levels were highest in the jejunum and ileum and reached their maximum on day 28. Our results suggest that the YSM plays a role in the degradation and uptake of the peptides that are digested by the activated yolk proteases, and it also functions in the lysosomal digestion of yolk lipoproteins. Furthermore, the proventriculus is possibly involved in the digestion of yolk proteins. Finally, the jejunum and ileum appear to be the primary sites for peptide digestion and absorption at the end of the incubation.

## Simple Summary

In the duck *Anas platyrhynchos domestica*, the mechanisms underlying the consumption of yolk proteins by the developing embryo are completely unexplored. In the present study, we investigated the function of the yolk sac membrane, proventriculus, and small intestine in the digestion and absorption of yolk proteins during the development of duck embryo. We found that the endodermal cells of the yolk sac membrane expressed genes which encode aminopeptidase N, cathepsin B, and cathepsin D proteases, and the oligopeptide transporter PepT1. The expression of these genes was reduced toward hatching. Furthermore, in the proventriculus, the expression of the gene, which produces embryonic duck pepsinogen protease, was largely decreased at later stages. In contrast, the small intestine expressed high levels of the genes that encode aminopeptidase N and the oligopeptide transporter PepT1 on the day of hatch. Our results suggest that before the last stages of development, the yolk sac membrane and proventriculus appear to function in protein digestion and the digested oligopeptides are probably absorbed by the yolk sac membrane cells. At the end of the incubation when the back flow of yolk into the small intestine occurs, the digested protein products are further degraded and taken up by the intestinal cells.

## 1. Introduction

Embryos of oviparous birds depend solely on egg nutrients for their development [1]. The two major sources of nutrients in avian eggs are egg white and egg yolk. In general, the egg white, before being consumed by the developing embryo, transfers through the amnion and embryo intestine into the egg yolk [2,3,4,5,6,7]. The egg yolk becomes progressively surrounded by the yolk sac membrane (YSM), which extends from the embryo hind gut [8,9]. Yolk proteins, which are primary nutrients for the embryo, appear to be degraded inside the egg yolk itself or in the endodermal cells of the YSM [10].

In the egg yolk, several classes of proteases were shown to be activated either before the major transfer of egg white into the yolk [11,12] or after it [6,7,13,14]. It has been proposed that the activated yolk proteases participate in the degradation of transferred egg white and endogenous yolk proteins [4,6,7,10,12,14]. The YSM also plays a major role in the processing of yolk proteins and lipoproteins. The membrane seems to have a dual function. One function is to continue the digestion and absorption of yolk proteins that are initially digested in the egg yolk. In support of this function, the endodermal cells of the YSM were found to express the gene that encodes aminopeptidase N, which is a membrane-bound zinc metalloprotease that cleaves amino acids from the N-terminus of peptides [7,9,15,16,17,18,19]. The cells were also shown to express membrane transporters for different types of amino acids and for the oligopeptide transporter PepT1, which is a proton-coupled transporter for di- and tripeptides [7,9,17,18,19,20,21]. Another function of the YSM is to uptake yolk lipoproteins into its endodermal cells using receptor-mediated endocytosis and to digest them by the lysosomes [21,22,23]. Indeed, the endodermal cells of the membrane were reported to express several genes related to the lysosomal digestion of lipids and lipoproteins, such as the genes that encode prosaposin, lipase A, and proteases, including cathepsin B, L2, A, and D [10,17,21].

The digested yolk components inside the endodermal cells of the YSM are used to synthesize new proteins and lipoproteins, which are delivered to the growing embryo by the blood vessels in the splanchnic mesoderm [17,21,22,23,24]. The delivered macromolecules contribute to building up different organ systems such as the digestive tract that grows from a primitive gut tube and differentiate into a complete tract, which includes the beak, esophagus, crop, stomach, small intestine, paired ceca, large intestine, and cloaca [25,26]. The stomach is composed of two distinct portions: the anterior glandular portion (proventriculus) and the posterior muscular one (gizzard) [25,27]. During development, the proventricular epithelium differentiates into luminal and glandular epithelia [25,28,29,30]. The glandular epithelium gives rise to the proventricular glands, which secrete an aspartyl protease known as embryonic pepsinogen [28,31]. The embryonic pepsinogen is only expressed during embryogenesis and is different from the adult pepsinogens which persist mainly throughout adult life [32,33].

The small intestine is composed of three major segments: the duodenum, jejunum, and ileum. During incubation, the growth of the small intestine is slow; however, rapid growth and maturation occur at the end of the incubation period [34,35]. During this period, the weight of the intestine relative to the embryo’s weight increases [18,36,37], and the absorption surface area of the intestine enlarges due to the increase in villus height and surface area, as well as the growth of new villi [36,38]. The morphological changes in the intestine are accompanied by changes in the expression of the genes that encode several brush border enzymes and nutrient transporters. Among these genes are aminopeptidase N (*APN*) and the oligopeptide transporter *PepT1*, which showed a high expression on the hatch day [18,19,39,40,41,42,43]. The morphological and gene expression changes in the small intestine at late developmental stages are necessary for the intestine to acquire the functional ability of digestion and absorption in order to accommodate the switch of feeding from yolk to external nutrient sources after hatching [18,36].

Although the consumption of egg nutrients by the developing embryo is largely explored in chicken [9,19,21,23,29,40] and turkey [14,39,41,44] that belong to the Galliformes order [45] and in pigeon [7,10,18,43], which is a member of Columbiformes [45], the nutrient consumption in Anseriformes is still largely undiscovered. Previously, in one species of Anseriformes, the duck *Anas platyrhynchos domestica* [45], we showed that an electrophoretic protein band with a molecular weight of 73.8 kDa disappeared from the duck egg yolk after incubation day 18. However, the intensity of most yolk protein bands, including those transferred from the egg white, was highly reduced on the hatch day [6]. Also, we found that almost all of the protein bands disappeared in the intestinal fluid on the day of hatch. Furthermore, we demonstrated that acidic aspartic proteases were activated late in development in both the egg yolk and intestinal fluid [6]. Accordingly, we suggested that the egg yolk proteins were initially degraded in the egg yolk by the activated proteases, but after the back flow of egg yolk into the small intestine on the day of hatch, the degradation of yolk proteins was completed in the intestine [6]. However, to the best of our knowledge, the role of the YSM in yolk protein processing during embryogenesis is completely unexplored in duck. Moreover, the function of the proventriculus in egg protein breakdown as well as the function of the small intestine in yolk protein digestion and absorption are still unknown. Here, we took a step toward understanding the mechanisms underlying the digestion and absorption of egg yolk proteins and lipoproteins during the development of duck embryo. To pursue our goal, we tested during different developmental stages in the YSM, the expression of cathepsin B (*CTSB*) and cathepsin D (*CTSD*), which encode lysosomal cysteine and aspartic proteases, respectively. Also, we examined at different stages the expression of embryonic duck pepsinogen (*EDPg*) in the proventriculus and the expression of *APN* and *PepT1* in the YSM and in the intestinal segments, which include the duodenum, jejunum, and ileum.

## 2. Materials and Methods

### 2.1. Egg Incubation and Embryo Staging

Fertilized eggs of *Anas platyrhynchos domestica* (Pekin duck breed) were purchased from local farms in Amman. They were collected immediately after laying and kept in an egg incubator (ELYE-3, Onelye Hk Group Limited, Nanjing, China) under the following conditions: 38.0 °C temperature, 60% humidity, and an automatic see-saw motion every 2 h. The developmental stages were measured by days, starting from the day of egg deposition (incubation day 0) and ending at the day of hatch (day 28).

### 2.2. Tissue Collection and Preparation

The YSMs, proventriculi, and small intestinal segments were collected from the fertilized eggs at developmental stages 12, 14, 16, 17, 18, 20, 22, 24, 26, and 28. Ninety fertilized eggs were used in total, and after the removal of undeveloped eggs or unrepresentative ones (i.e., the ones that had delayed growth), five to six eggs were analyzed per stage. The egg was opened at its pointed end to facilitate the removal of allantoic, extraembryonic, and amniotic fluids, if present, as these fluids reside at the upper end of the egg. Then, the embryo and attached yolk sac (YS), which is composed of yolk contents surrounded by the YSM [9], were decanted in a clean bowl. A hole, 0.5 cm in diameter, was made in the YS, and the yolk was sucked using a 5 mL syringe without a needle. Following that, the YSM was transferred to a sterile Petri dish, cut open using a sterile blade, washed several times with diethyl pyrocarbonate (DEPC)-treated distilled water, and immediately used for RNA extraction as described below. The absorption of the YS by the embryo abdomen started on incubation day 24 and completed on day 28 [46]. Therefore, during these stages, a hole was made in the embryo abdomen in order to pull the YS outside the body.

The embryo was transferred to a sterile Petri dish and dissected under an Olympus SZ4045 Stereo Microscope (Spectra Services, Inc., Ontario, NY, USA). The whole digestive tract was removed, washed with DEPC-treated water, and briefly dried. Then, the proventriculus, which is located below the esophagus and above the gizzard [47], was excised and collected. After that, the three different components of the small intestine, including the duodenum, jejunum, and ileum, were collected. The duodenum is the proximal portion of the small intestine that comes after the gizzard and forms a “U” shaped structure around the pancreas [47]. It is followed by the jejunum, which is separated from the ileum by Meckel’s diverticulum [40]. The ileum extends posteriorly and ends at the ileo-cecal-colic junction where the two ceca arise [47]. The proventriculus, duodenum, jejunum, and ileum were cut open longitudinally, washed with DEPC-treated water, and immediately processed for RNA extraction.

### 2.3. RNA Extraction, cDNA Synthesis, and Gene Expression Analysis

Total RNA was extracted from the tissues of YSMs, proventriculi, duodena, jejuna, and ilea during different developmental stages using 1 mL of TRIzol reagent (Ambion, Carlsbad, CA, USA) per 100 mg of each tissue based on the manufacturer’s protocol. Following DNase-1 treatment and phenol/chloroform purification, the RNA extracts were reversed-transcribed to cDNAs using the ProtoScript First Strand cDNA Synthesis Kit (New England Biolabs, Ipswich, MA, USA). Briefly, 1 µg of each RNA extract was reversed-transcribed by M-MuLV enzyme at 42 °C for 1 h using oligo (dT) adaptor primer according to the manufacturer’s procedure. The synthesized cDNAs were then used to perform reverse transcription–quantitative polymerase chain reaction (RT-qPCR) using the LineGene 9600 PLUS Real-Time PCR Detection System (Bioer Technology, Hangzhou, China) to determine the expression levels of *APN*, *PepT1*, *CTSB*, *CSTD*, and *EDPg* genes during different developmental stages. *β-actin*, 28S ribosomal RNA (*28S rRNA*), and glyceraldehyde 3-phosphate dehydrogenase (*GAPDH*) genes were used as internal controls, to which the fold changes in gene expression were normalized. RT-qPCR was carried out in a 20 µL reaction using 1.5 µL of cDNA, 10 µL of Luna Universal qPCR Master Mix (New England Biolabs, Ipswich, MA, USA), and gene-specific primers at a final concentration of 0.6 mM (Table 1). The PCR conditions used were 50 °C for 2 min, 95 °C for 2 min, and 40 cycles of 95 °C for 15 s, 59 °C for 15 s, and 72 °C for 1 min. The specificity of amplification was verified by performing melting curve analysis under the following conditions: 95 °C for 15 s, 60 °C for 1 min, and 95 °C for 15 s. The mean of the relative expression of each gene at each stage was calculated from four different experiments using four different pools of cDNA, and each cDNA was read in duplicate. Stage 12 was used as a reference stage during quantification. Fluorescence emission was detected, and relative quantification was calculated automatically by the software of LineGene 9600 PLUS Real-Time PCR Detection System (FQD-96a V1.0).

### 2.4. Statistical Analysis

Data were analyzed using IBM SPSS (Statistical Package for Social Sciences) statistics version 21 (Chicago, IL, USA). One-way analysis of variance (ANOVA) was used to assess the presence of any significant differences among the means of relative gene expression of *APN*, *PepT1*, *CTSB*, and *CTSD* in the YSM and *EDPg* in the proventriculus during different developmental stages. The one-way ANOVA was followed by Tukey’s Post Hoc test for multiple comparisons to determine the means of relative gene expression that differ significantly from each other during different stages. The F-value in the one-way ANOVA was calculated according to the following formula: F (df_between_, df_within_) = MS_b_/MS_w_, where df_between_: degree of freedom between groups, df_within_: degree of freedom within groups, MS_b_: mean square between groups, MS_w_: mean square within groups. The main effect of the intestinal segment and of the developmental stage and their interaction on the means of *APN* and *PepT1* relative gene expression were tested by a two-way ANOVA using the General Linear Model (GLM). The two-way ANOVA was followed by Tukey’s Post Hoc test for pairwise comparisons to identify the means of relative gene expression that differ significantly from each other in different intestinal segment and the means that differ at different stages. The Bonferroni test was used to adjust the confidence intervals in pairwise comparisons between the means of relative gene expression under the interaction effect of segment and stage. The F-value in the two-way ANOVA was calculated as the following: F (df_factor_, df_error_) = MS_f_/MS_e_, where df_factor_: degree of freedom of factor (main or interaction effect), df_error_: degree of freedom of error, MS_f_: mean square of factor, MS_e_: mean square of error. The level of significance in all the statistical tests was set at *p* ≤ 0.05.

## 3. Results

### 3.1. Gene Expression in the YSM During the Development of Duck Embryo

**Figure 1:**
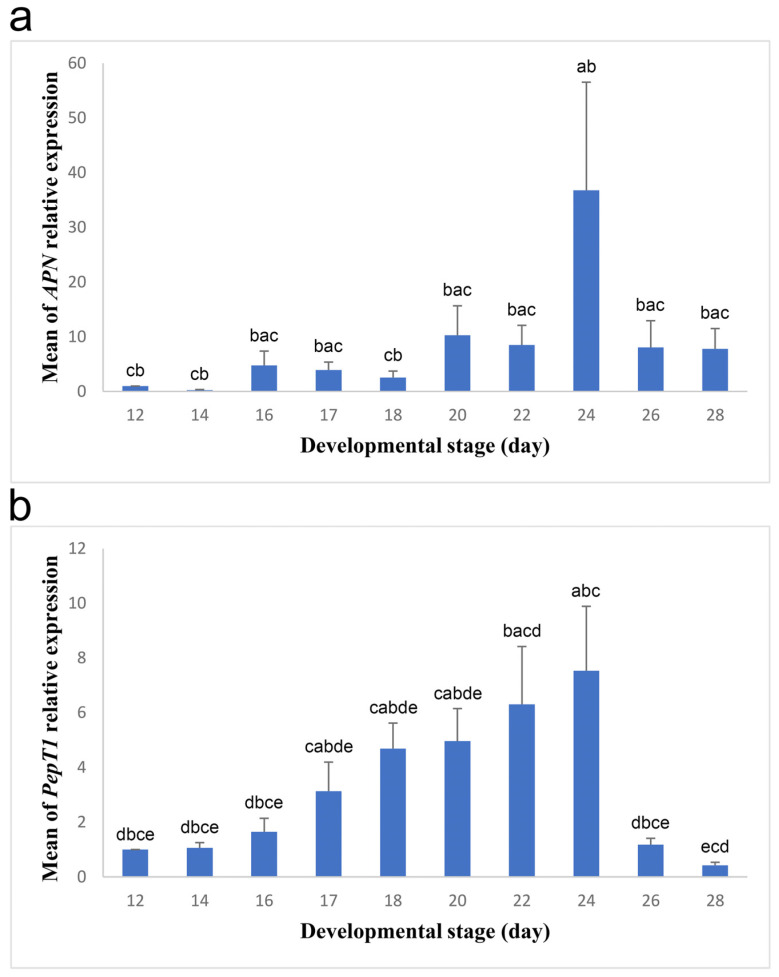
Relative expression of *APN* and *PepT1* in the YSM during different developmental stages. Blue bars represent the means of *APN* (**a**) and *PepT1* (**b**) relative expression at different stages, while black lines mark the standard errors of the means. The F- and *p*-values of one-way ANOVA are F (9, 30) = 2.304, *p* = 0.042 and F (9, 30) = 4.628, *p* = 0.001 for *APN* and *PepT1* expression, respectively. Bars with different letters (a–e) differ significantly in the means of relative gene expression.

To uncover the dual function of the duck YSM during embryogenesis, we started by exploring its function in the further degradation and uptake of the peptides which resulted from the initial breakdown of egg proteins inside the egg yolk [6]. To accomplish that, we examined the expression of *APN* and *PepT1* genes in the membrane during development (Figure 1). *APN* expression was initially low, and then it began to increase between days 16 and 22, but with a reduction on day 18 (Figure 1a). After that, the expression peaked and reached the maximum value on day 24; however, it decreased thereafter. The expression on day 24 was significantly larger than that on days 12, 14, and 18 (Figure 1a). *PepT1* expression was low on incubation days 12 to 16 (Figure 1b). Then, it increased in the period between days 17 and 20, became further high on day 22, and reached the highest value on day 24. After that, the expression dramatically decreased on day 26 and reached the lowest value on day 28. The highest expression on day 24 was substantially higher than the expression on days 12 to 16, 26, and 28, while the lowest expression on day 28 was considerably lower than the expression on days 22 and 24 (Figure 1b).

**Figure 2:**
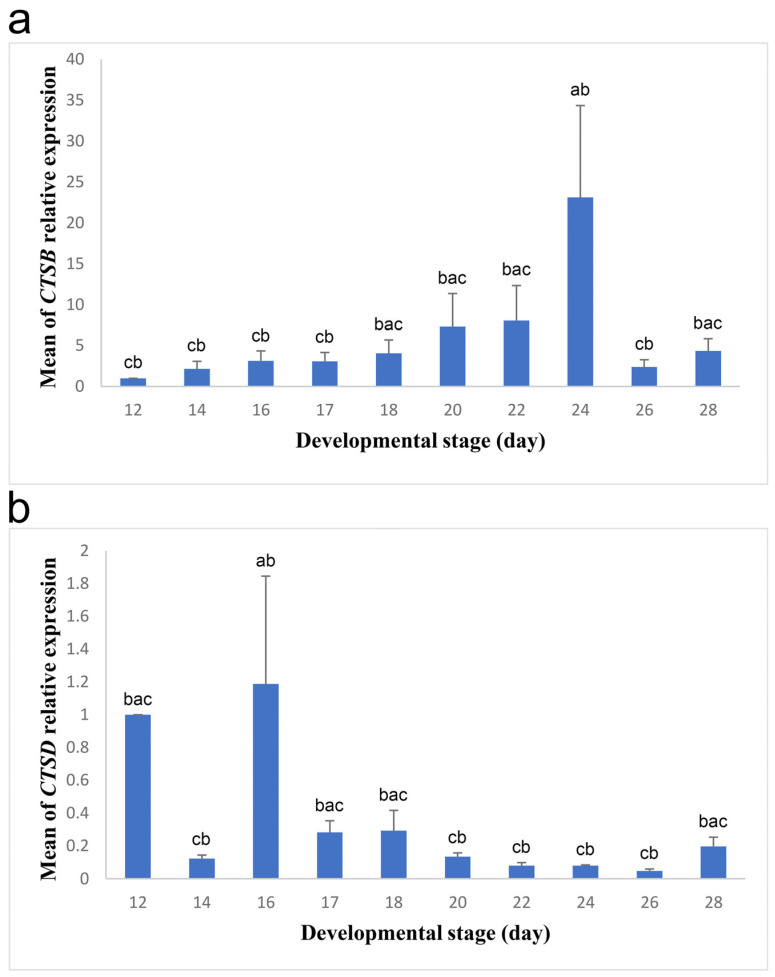
Relative expression of *CTSB* and *CTSD* in the YSM during different developmental stages. Blue bars represent the means of *CTSB* (**a**) and *CTSD* (**b**) relative expression at different stages, while black lines mark the standard errors of the means. The F- and *p*-values of one-way ANOVA are F (9, 30) = 2.445, *p* = 0.032 and F (9, 30) = 3.615, *p* = 0.004 for *CTSB* and *CTSD* expression, respectively. Bars with different letters (a–c) differ significantly in the means of relative gene expression.

Then, we investigated the role of YSM cells in the digestion of yolk lipoproteins. To achieve that, we tested the expression of *CTSB* and *CTSD* in the membrane from incubation day 12 to day 28 (Figure 2). In general, the expression of *CTSB* was higher than that of *CTSD* throughout development (Figure 2). This suggests that the encoded cysteine protease cathepsin B and not the aspartic protease cathepsin D plays a main role in the lysosomal digestion of yolk lipoproteins that are endocytosed by the endodermal cells of the YSM. We found that the expression of *CTSB* was low during the early examined stages between days 12 and 17 (Figure 2a). Then, it started to increase significantly on days 18 to 22 and reached the maximum on day 24. Following that, the expression drastically decreased on day 26 and then increased, though not largely, on day 28. The maximum expression was obviously larger than the expression in the early stages and the expression on day 26 (Figure 2a). *CTSD* showed a different expression pattern compared to that of *CTSB* as the largest expression values of the gene were detected in the early stages (Figure 2). Thus, if cathepsin D plays a role in lipoprotein degradation, albeit minor, it will be in the early stages. *CTSD* expression was high on day 12; however, it decreased on day 14 and then abruptly increased and reached the highest value on day 16 (Figure 2b). After that, the expression decreased on days 17 and 18 and became even lower on days 20 to 26. On the day of hatch (day 28), the expression increased, although not by much. The minimum expression on day 14 and from days 20 to 26 was significantly lower than the expression on day 16 (Figure 2b).

### 3.2. Gene Expression in the Digestive Tract During the Development of Duck Embryo

**Figure 3:**
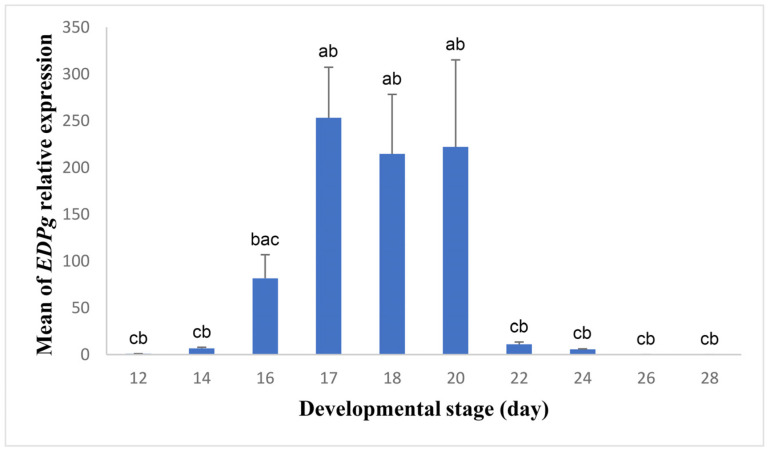
Relative expression of *EDPg* in the proventriculus during different developmental stages. Blue bars represent the means of *EDPg* relative expression at different stages, while black lines mark the standard errors of the means. The F- and *p*-values of one-way ANOVA are F (9, 30) = 7.034, *p* = 0.000. Bars with different letters (a–c) differ significantly in the means of relative gene expression.

To uncover the role of the digestive tract in egg protein consumption, we explored the functions of the proventriculus and small intestine during the development of duck embryo. We began with the proventriculus and tested the expression of *EDPg* to elucidate whether or not the proventriculus functions in the initial digestion of egg proteins (Figure 3). The expression of *EDPg* was low on days 12 and 14. Then, it started to increase significantly on day 16 and became considerably high and reached the maximum values in the period between days 17 and 20. Following that, the expression dropped tremendously and remained low toward hatching. The period of maximum expression was significantly higher than the expression on days 12 and 14 and the expression from days 22 to 28 (Figure 3).

**Figure 4:**
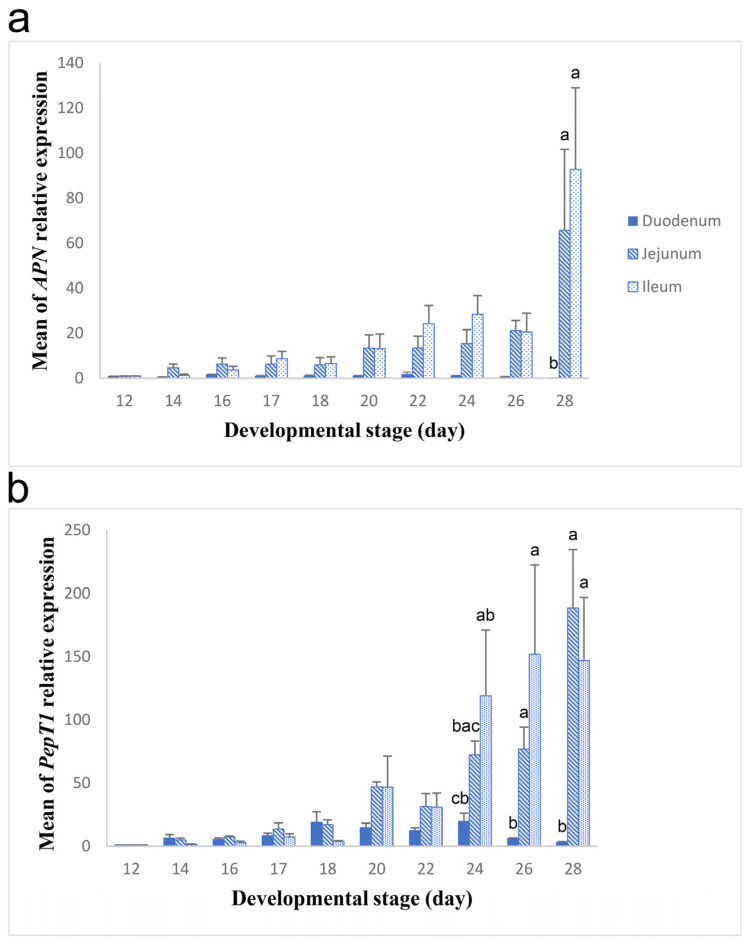
Relative expression of *APN* and *PepT1* in the small intestine during different developmental stages. The means of relative expression of *APN* (**a**) and *PepT1* (**b**) in the duodenum, jejunum, and ileum at different stages are marked by filled, striped, and dotted blue bars, respectively. Black lines mark the standard errors of the means. The interaction of segment and stage showed a significant effect on the expression of *APN* (*p* = 0.017) and *PepT1* (*p* = 0.000) among the duodenum, jejunum, and ileum at certain developmental stages. Bars with different letters (a–c) differ significantly in the means of relative gene expression among the intestinal segments at specific stage.

Following that, we investigated the role of the small intestine in the additional digestion and absorption of the degraded protein products by examining during embryogenesis the expression of *APN* and *PepT1* in different intestinal segments, including the duodenum, jejunum, and ileum. The intestinal segment and the developmental stage as two main effects as well as their interaction significantly affected the expression of the two genes (Table 2). The expression of *APN* in the duodenum was obviously lower than that in the jejunum and ileum (Table 2, segment factor, *p* = 0.000). Moreover, the expression of the gene was generally low throughout development; however, it increased dramatically on day 28 (Table 2, stage factor, *p* = 0.000). On day 28, the *APN* expression showed spatial differences among the intestinal segments. It was significantly higher in the jejunum and ileum compared to that in the duodenum (Figure 4a; Table 2, segment x stage factor interaction, *p* = 0.017). Similarly, the expression of *PepT1* was prominently lower in the duodenum compared to its expression in the jejunum and ileum (Table 2, segment factor, *p* = 0.000). Also, the largest expression of the gene was on day 28. The expression changed little in the period between days 12 and 17. Then, it started to increase significantly on day 18, and this increase was generally gradual to the hatch day (Table 2, stage factor, *p* = 0.000). The *PepT1* expression differed significantly among the intestinal parts on days 24 to 28 (Figure 4b; Table 2, segment x stage factor interaction, *p* = 0.000). *PepT1* expression on day 24 was lowest in the duodenum, highest in the ileum, and intermediate in the jejunum (Figure 4b). The expression on day 26 and day 28 was also significantly low in the duodenum; however, it was strongly high in both the jejunum and ileum (Figure 4b).

## 4. Discussion

In the present study, we took a step toward exploring the mechanisms underlying the consumption of egg yolk proteins and lipoproteins by the developing duck embryo. We found that (1) in the YSM during embryogenesis, the expression of *CTSB* was higher than that of *CTSD*, and the expression of both *APN* and *PepT1* peaked on day 24; (2) in the proventriculus, the expression of *EDPg* reached its maximum value in the period between incubation days 17 and 20; and (3) in the intestinal segments, *APN* expression was abruptly increased on the hatch day, while *PepT1* expression became obviously high after day 22. For both genes, the expression was lowest in the duodenum.

The YS is the main source of nutrients for the developing avian embryo before the back flow of yolk content from the YS into the small intestine through the yolk stalk, which occurs slightly before hatching [9,18,48]. Previously, we showed that the yolk proteins were degraded inside the egg yolk possibly by the activated acidic aspartic proteases late in development [6]. Therefore, in this study, we explored the role of the YSM in the additional processing of degraded protein products by testing the expression of *APN* and *PepT1* genes in the membrane. The expression of *APN*, in general, began to increase in the period between days 16 and 22 and reached the highest value on day 24. The increase in the level of expression nearly coincided with the time of egg yolk protein degradation which started after incubation day 18 [6]. This suggests that the encoded aminopeptidase N continues the digestion of the peptides that resulted from the initial degradation by yolk proteases. The expression of *PepT1* increased in the period between days 17 and 22 and became maximum on day 24. The high level of expression was also concurrent with the yolk protein degradation [6] and the increase in *APN* expression. The encoded transporter probably participates in the transfer of the degraded di- and tripeptides into the endodermal cells of the YSM.

The expression of *APN* and *PepT1* was also examined in the chicken [9,19] and pigeon [7,18] YSM during embryogenesis. The expression of both genes in chicken increased from incubation days 11 to 15 and decreased thereafter [19]. The expression patterns of *APN* and *PepT1* in chicken were similar to their corresponding genes in duck, as the expression of the two genes increased until reaching the maximum value and following that decreased ([19]; this study). In pigeon, *APN* expression was high at first and then generally decreased toward the end of incubation [7]. *PepT1* expression increased gradually, reached the maximum values in the period between days 13 and 15, and decreased thereafter [7]. The pigeon *APN* expression pattern does not match with that of duck as the level of expression in pigeon was initially at the maximum [7], while the highest value in duck was reached with the progress of development. Also, *PepT1* expression in pigeon showed a period of maximum expression [7], whereas the increase in *PepT1* expression in duck was gradual until reaching the highest value. Nevertheless, in both species, the expression of the two genes decreased toward the day of hatch ([7]; this study).

Following that, we investigated the function of the YSM in the lysosomal digestion of yolk lipoproteins by examining the expression of *CTSB* and *CTSD* in the membrane during development. It appears that cathepsin D plays a secondary role in digestion because the expression of the *CTSD* gene was weak all over the examined stages. *CTSB* expression was low before incubation day 18, and then it increased in the period between days 18 and 22 and peaked on day 24. The dynamic changes in the expression of the gene may reflect the dynamic changes in the uptake of lipoproteins by receptor-mediated endocytosis. In chicken, the expression of the major lipoprotein transport receptor known as LR8 (low-density lipoprotein receptor relative to eight ligand binding repeats) varied according to the embryonic stage [22]. Furthermore, the expression level of the lipoprotein receptor triad, LRP2 (low-density lipoprotein receptor-related protein-2)–cubilin–amnionless, changed significantly with the progressive vascularization of the membrane [23]. Although, as far as we know, the receptor and/or receptor complex responsible for the uptake of lipoproteins by the endodermal cells of the duck YSM are still unexplored, it is possible that the expression of presumed receptors in the early studied stages is low. This may cause a reduction in the level of endocytosed lipoproteins. As a consequence, the lysosomal digestion becomes minimal and thus the expression of *CTSB* is reduced. With the progress of embryonic development and the increase in the vascularization of the membrane, the expression of the receptors may increase. This may result in the increase in *CTSB* expression to participate in the digestion of internalized lipoproteins. It is unknown, however, whether the other lysosomal cysteine proteases like cathepsin C and L [49] and serine proteases such as cathepsin A and G [49] also participate in lipoprotein digestion in the duck YSM. Future studies are awaited to uncover the action of these lysosomal proteases in the YSM of duck embryo.

The expression of cathepsins in the YSM was also studied in other avian species. In chicken, Yadgary et al. (2014) performed a transcriptome study of the YSM on embryonic days 13, 15, 17, 19, and 21 (the day of hatch) [21]. They found that among the 50 highest expressed genes during the examined stages were cathepsin A (*CTSA*) on day 13, cathepsin L2 (*CTSL2*) on days 13, 15, 19, and 21, and *CTSB* on days 15 to 21 [21]. The expression of *CTSB* in the YSM was high at the end of incubation [21], which is inconsistent with our findings in the duck YSM where *CTSB* expression seemed to be reduced toward hatching. In pigeon, consistent with our findings in duck, we showed that *CTSB* appeared to play a primary role in yolk lipoprotein digestion, whereas *CTSD* seemed to play a minor role as the expression of the gene was weak and fluctuated insignificantly throughout development [10]. The expression pattern of *CTSB* in the pigeon YSM is almost in agreement with that in duck because after the gradual increase, the maximum value was reached on incubation day 13, and then the expression was reduced toward the day of hatch (day 17) [10].

Taken together, our results elucidate that the general pattern for the expression of *CTSB*, *APN*, and *PepT1* in the duck YSM is reduction after day 24 which continued toward hatching. The decrease in the expression of these genes largely coincided with the internalization of the YS into the embryo abdomen which started on day 24 and completed on day 28 [46]. This period was also marked by the onset of YSM degradation [46], probably due to the induction of apoptosis [50]. The YS of chicken embryo was also shown to internalize and start degradation at the end of incubation [51]. Reno et al. (2022) demonstrated that the post-hatch degradation of the chick YS was triggered by apoptosis [50]. Furthermore, the reduction in the expression of genes in the duck YSM on the day of hatch was concurrent with the back flow of yolk into the intestinal lumen through the yolk stalk [6]. Thus, the major route of yolk transfer into the embryo is shifted from the YSM to the yolk stalk [48], and as a consequence, the expression of YSM genes is likely no longer required. The shift in the route of yolk transfer was suggested to occur on incubation days 20 to 21 in chicken [9] and incubation days 16 to 17 in pigeon [18]. Similarly, previous studies have shown that the expression of the digestive and transporter genes was downregulated toward the end of incubation in the YSM during the shift period [9,18].

The YS and digestive tract are associated structures as the YSM extends from the hind gut [8,9]. Therefore, it is reasonable to assume that the digestive tract also plays a role in egg protein processing during embryogenesis. To test our assumption, we began with the proventriculus and examined the expression of *EDPg* during the development of duck embryo. We found that the expression of the gene was low on incubation days 12 and 14. Previous studies in chicken showed that the proventricular glands, which secrete embryonic chicken pepsinogen (*ECPg*) [31], were initially identified as simple unbranched glands on embryonic day 7 [30]. Northern blot analysis showed that the mRNA level of *ECPg* at this stage was barely detectable [31]. Similarly, the low expression of *EDPg* during the early examined stages can be due to the onset of the formation of the proventricular glands in the duck embryo that are probably less differentiated and express low levels of the gene during these stages. Then, we showed that the expression of *EDPg* began to increase significantly on incubation day 16 and peaked in the period between days 17 and 20. In chicken, the mRNA level of *ECPg* increased after embryonic day 8 and became most abundant on day 15 [31,33]. The high mRNA level of the gene coincided with the progressive growth and differentiation of the chicken proventricular glands, which became branched and acquired complex structures [30]. Thus, the increase in *EDPg* expression may result from the increase in the development and differentiation of the duck proventricular glands such that their cells express high levels of the gene. In addition, the period of high *EDPg* expression was correlated with the chronological transfer of egg white, which started on incubation day 17 in the amniotic fluid and intestinal lumen and on day 19 in the egg yolk [6]. It is possible that the passage of egg white through the proventriculus, on its way toward the intestine and egg yolk, induces the proventricular glands to express high levels of *EDPg*. The encoded protein, however, does not apparently have any digestive effect before it reaches the egg yolk. This can be inferred from the electrophoretic pattern of the intestinal fluid which did not show any sign of egg white protein degradation before the hatch day [6]. The embryonic duck pepsinogen may remain inactive due to the presence of a coexisting inhibitor [2] or environmental conditions such as changes in pH [4]. In quail, it was also shown that the specific activity of embryonic pepsinogen peaked on incubation days 12 and 13 [52]; however, the digestion of transferred egg white proteins in the intestinal lumen occurred on day 16, one day before hatch [4]. Once pepsinogen reaches the egg yolk together with the transferred egg white proteins, it may become activated into pepsin. It is possible that the aspartic acidic proteases, which were activated late in development in the duck egg yolk [6], included the activated pepsin.

Following the peak of *EDPg* expression, we elucidated that the expression of the gene drastically decreased, and the decrease continued toward the end of incubation. The reduction in *EDPg* expression may reveal the silencing of the gene probably by the epigenetic mechanisms in preparation for the expression of adult duck pepsinogen genes. In chicken, the expression of *ECPg* decreased rapidly after incubation day 18 [31,33]. Moreover, during the last stages of development, the expression of two adult pepsinogen genes was detected: chicken pepsinogen A (*cPgA*) on incubation days 16 to 19 and chicken pepsinogen C (*cPgC*) on incubation days 17 and 18 [33]. Then, the expression of the two genes disappeared temporarily but appeared again one day after hatching and increased rapidly thereafter [33]. However, to our knowledge, the expression of adult duck pepsinogen genes around the day of hatch is still unexplored. Thus, the temporal switch between the expression of *EDPg* and the expression of adult pepsinogen genes cannot be predicted. Prospective studies can help reveal the temporal changes in the expression of adult genes.

After that, we investigated the function of the small intestine in the additional processing of egg proteins. Therefore, we tested the expression of *APN* and *PepT1* in the intestinal segments during development. We found that the intestinal segment and developmental stage as well as their interactions affected the expression of the two genes. According to the intestinal segment effect, the expression of both *APN* and *PepT1* was highest in the jejunum and ileum and lowest in the duodenum. Our results suggest that the jejunum and ileum are the primary sites for peptide digestion and absorption during incubation. Based on the stage effect, we demonstrated that *APN* expression was low throughout development; however, it increased abruptly on the hatch day when the back flow of yolk into the intestinal lumen occurred [6]. Previously, we showed that almost all of the electrophoretic protein bands disappeared in the intestinal fluid on the day of hatch. We also found that the activated proteases in the intestinal lumen belonged to an acidic aspartic class of proteases [6]. One possibility is that the aspartic proteases are transferred on the day of hatch from the egg yolk into the intestine along with other yolk proteins, and they continue the digestion of the back-flowed proteins in the intestine [6]. Another possibility, supported by our present study, is that the pH conditions inside the intestinal lumen may become alkaline on the day of hatch due to pancreatic secretions. In this case, the encoded aminopeptidase N, which is a metallopeptidase [16], is likely activated and continues the digestion of the back-flowed peptides in the intestine. Furthermore, we showed that the expression of *PepT1* started to increase gradually on day 18 and became maximum on the hatch day. The increase in the expression before the day of hatch may reveal the progressive functional maturation of the intestine at late developmental stages in preparation for the post-hatch period when the ducklings depend on exogenous nutrients. The maximum expression on the day of hatch probably reflects the great need for the production of a high level of the encoded oligopeptide transporter to transport the oligopeptides after the additional digestion of the back-flowed degraded protein products. Finally, the interaction effect of the segment and stage illustrated that the jejunum and ileum showed high levels of *APN* and *PepT1* expression at the end of the incubation or at late stages, respectively, whereas the duodenum showed low levels of the expression of both genes. It is possible that the back-flowed yolk is transferred to the upper portion of the intestine (duodenum) by the anti-peristaltic contractions of the small intestine [36], where the digestion of egg yolk proteins is probably continued by the pancreatic enzymes [36] and not by the duodenal aminopeptidase N. Following that, the digested peptides are further processed in the jejunum and ileum by the products of highly expressed *APN* and *PepT1* genes.

The expression of *APN* and *PepT1* in the three intestinal segments was also investigated during the development of other avian embryos. *APN* expression in chicken [40] and pigeon [18] was highest in the ileum, lowest in the duodenum, and intermediate in the jejunum. The expression of *APN* in different intestinal segments of chicken and pigeon is consistent with that in duck, except for the expression in the jejunum, which was intermediate in the two species [18,40], while *APN* expression was maximum in both the jejunum and ileum in duck. Furthermore, *APN* expression in the small intestine increased from embryonic day 20 to the day of hatch in chicken [40], and it was upregulated from incubation day 12 toward the end of incubation (day 17) with a decline on day 16 in pigeon [18]. *APN* expression increased on the day of hatch in chicken [40], pigeon [18], and duck. However, the pattern of expression differed between the pigeon and duck. *APN* expression in pigeon is marked by an increase, followed by a decrease one day before hatching, and then an increase [18], while *APN* expression in duck was low and changed little during development but suddenly increased at hatching. With respect to *PepT1* expression, it was highest in the duodenum, lowest in the ileum, and intermediate in the jejunum in chicken [40]. In pigeon, the expression was maximum in the duodenum and jejunum and minimum in the ileum [18]. The spatial expression of *PepT1* in chicken [40] does not correspond with that in duck, whereas only the highest expression in the jejunum of pigeon [18] is consistent with that in duck. Moreover, the expression of *PepT1* in chicken small intestine increased from embryonic day 10 to the end of the incubation [37]. The expression in pigeon changed little on embryonic days 9 to 13 and then increased significantly from incubation day 15 to the day of hatch [43]. The mRNA level of *PepT1* in turkey small intestine increased quadratically with age from embryonic day 23 (E23) to the hatch day (d0) [39]. The expression was barely detectable on E23 but increased 3.2-fold from E23 to d0 [39]. The temporal expression of *PepT1* in pigeon is consistent with that in duck because the period of little change in gene expression was followed by a general increase in the expression which continued toward hatching ([43]; this study). On the other hand, the expression in chicken partially matched that in duck, whereas the expression in turkey did not, as the expression increased gradually in chicken [37] and non-linearly in turkey [39]. Taken together, the results from previous studies [18,37,39,40,43] and from this work show that the expression of *APN* and *PepT1* in the small intestine of developing avian embryo is upregulated on the hatch day; however, there are species-, tissue-, and stage-related differences.

## 5. Conclusions

In conclusion, the present study is the first to investigate the role of the YSM and digestive tract in the consumption of yolk proteins during the development of duck embryo. Our results suggest that the YSM has a dual function. The YSM continues the digestion and absorption of the peptides that are initially degraded in the egg yolk itself by the activated proteases. Moreover, it carries out the lysosomal digestion of the lipoproteins that are taken up by the endodermal cells of the membrane using receptor-mediated endocytosis. The digestive tract also appears to play an important role in egg protein processing. The proventriculus is possibly involved in the digestion of yolk proteins. Furthermore, the jejunum and ileum of the small intestine seem to continue the digestion and absorption of the egg yolk peptides after the back flow of egg yolk into the small intestine on the day of hatch. Future studies should examine the developmental roles of the YSM and digestive tract in other species that belong to Anseriformes. This will help to better understand whether or not the same mechanisms of egg protein processing have evolved in this order.

## Figures and Tables

**Table 1:** Gene specific primers that were used in RT-qPCR.

Gene	GenBank Accession Number	Primer Sequence (5′-3′)	Product Size (bp)
*APN*	XM_027466576.2	F: 5′-CTTTGCCTCCTACGTGGAATAC-3′	97
		R: 5′-CATCACGGTGTAGAGTTCGTTC-3′	
*PepT1*	NM_001310803.1	F: 5′-CAGTGGCTGTCGGTAACATAA-3′	98
		R: 5′-AAACAGCAAGGCAGCAAAC-3′	
*CTSB*	XM_038176845.1	F: 5′-AGAAGCTCTGTGGCACTTTC-3′	99
		R: 5′-GCTTCCGTGAGTCGAAGTTATC-3′	
*CTSD*	XM_027457884.2	F: 5′-GGGACCCATAAGGAACATCAAG-3′	114
		R: 5′-CACCGAGCCTGTCTGAATTT-3′	
*EDPg*	XM_038168510.1	F: 5′-CATGCCTGATGTTGTCTTTGTC-3′	98
		R: 5′-AACCACTGATACACGCTTCTT-3′	
*β-actin*	NM_001310421.1	F: 5′-CCAAAGCCAACAGAGAGAAGA-3′	137
		R: 5′-ATCACCAGAGTCCATCACAATAC-3′	
*28S rRNA*	XR_003493880.2	F: 5′-GTAAACGGCGGGAGTAACTATG-3′	98
		R: 5′-GACAGTGGGAATCTCGTTCATC-3′	
*GAPDH*	XM_038180584.1	F: 5′-GCCATTCCTCCACCTTTGAT-3′	102
		R: 5′-CACGGTTGCTGTATCCATACTC-3′	

**Table 2:** The effect of intestinal segment, developmental stage, and their interaction on gene expression.

Factor	Gene Expression ^#^	
Segment	*APN*	*PepT1*
Duodenum	1.017 ± 0.118 ^b^	9.590 ± 1.442 ^b^
Jejunum	15.309 ± 4.387 ^a^	49.186 ± 9.772 ^a^
Ileum	20.021 ± 5.423 ^a^	51.499 ± 13.243 ^a^
*p*-value	0.000	0.000
F-value	F (2, 90) = 9.707	F (2, 90) = 12.570
**Stage**		
12	1.000 ± 0.000 ^b^	1.000 ± 0.000 ^fde^
14	2.121 ± 0.768 ^b^	4.223 ± 1.167 ^fde^
16	3.856 ± 1.128 ^b^	5.153 ± 0.794 ^fde^
17	5.341 ± 1.751 ^b^	9.803 ± 1.935 ^fde^
18	4.575 ± 1.517 ^b^	14.942 ± 3.691 ^ecdf^
20	9.123 ± 3.186 ^b^	36.246 ± 8.825 ^dbcef^
22	13.126 ± 4.026 ^b^	28.904 ± 6.409 ^dbcef^
24	14.996 ± 4.584 ^b^	70.368 ± 20.226 ^cabde^
26	14.093 ± 4.077 ^b^	84.080 ± 27.711 ^bacd^
28	52.925 ± 19.339 ^a^	112.865 ± 31.551 ^abc^
*p*-value	0.000	0.000
F-value	F (9, 90) = 6.879	F (9, 90) = 10.391
**Segment x Stage ^##^**		
*p*-value	0.017	0.000
F-value	F (18, 90) = 2.012	F (18, 90) = 3.200

**^#^** Gene expression is represented as the mean of relative expression ± standard error of the mean (SEM). ^(a–f)^ Different superscripts within the same column indicate significant difference in gene expression. **^##^** Represents the interaction effect of the intestinal segment and developmental stage on gene expression. The *p*- and F-values were calculated by two-way ANOVA using GLM. The values were considered significant at *p* ≤ 0.05.

## Data Availability

The original contributions presented in the study are included in the article, further inquiries can be directed to the corresponding author.
